# Preparation of Cationic Polyacrylamide Suspension and Its Application in Oilfield Wastewater Treatment

**DOI:** 10.3390/polym16010151

**Published:** 2024-01-03

**Authors:** Zhongjin Wei, Wenjun Long, Shaohua Li, Yu Zhao, Siting Yu, Fengshan Zhou

**Affiliations:** Engineering Research Center of Ministry of Education for Geological Carbon Storage and Low Carbon Utilization of Resources, Beijing Key Laboratory of Materials Utilization of Nonmetallic Minerals and Solid Wastes, National Laboratory of Mineral Materials, School of Material Sciences and Technology, China University of Geosciences, Beijing 100083, China; 3003190032@cugb.edu.cn (Z.W.); longwj@cugb.edu.cn (W.L.); 2103210080@email.cugb.edu.cn (S.L.); 2103210079@email.cugb.edu.cn (Y.Z.); 2103210078@email.cugb.edu.cn (S.Y.)

**Keywords:** cationic polyacrylamide, flocculant, suspension, oilfield wastewater

## Abstract

Cationic polyacrylamide (CPAM) solid particle is one of the most commonly used organic polymer flocculants in oilfield wastewater treatment, but it poses some problems, such as a slow dissolution rate and an easy formation into a “fish-eye” in the process of diluting into aqueous solution. However, the current liquid CPAM products also have some problems, such as low effective content, poor storage stability, degradation in a short time, and high preparation costs. In this paper, a CPAM suspension was successfully prepared with 50.00% CPAM fine powder, 46.87% oil phase solvent, 0.63% separating agent, 1.56% emulsifying and dispersing agent, and 0.94% rheology modifier. This suspension has an effective content of 50.00%. It also showed no separation in 7 days of storage at room temperature, no separation in 30 min of centrifugation at a speed of 2000 rpm, and diluted to a 0.40% solution in just 16.00 min. For 1000 NTU of diatomite-simulated wastewater, the optimal turbidity removal rate of the suspension was 99.50%, which was higher than the optimal turbidity removal rate of 98.40% for the inorganic flocculant polymeric aluminum chloride (PAC). For oilfield wastewater, the optimal turbidity removal rate of the CPAM suspension was 35.60%, which was higher than the optimal turbidity removal rate of 28.40% for solid particle CPAM. In a scale-up test, the CPAM suspension achieved a good application effect.

## 1. Introduction

Oilfield wastewater mainly comes from oilfield extraction water, drilling fluid waste fluid, refinery wastewater, etc. After mixing, its composition is very complex, and its main characteristic is that it contains a large number of oil droplets of different sizes and suspended matter [1,2,3,4]. The treatment methods of oilfield wastewater mainly include the physical separation method, biological treatment method, and chemical flocculation method [5]. The physical separation method is mainly used to remove oil and suspended solids from oily wastewater, etc. The main treatment processes are the gravity separation process, membrane separation process, air flotation process, and so on. The physical separation method has the advantages of simple operation and low cost, but there are obvious shortcomings including that the separation is not thorough enough [6]. The biological treatment method employs microorganisms in some wastewater organic matter decomposition into some relatively simple substances, mainly used in the treatment of oilfield wastewater with high water quality requirements; the disadvantage of the biological method is that it needs to be based on the characteristics of the sewage for the cultivation of special biological strains of bacteria, and sewage water quality changes need to be cultivated to produce different strains of biological bacteria [7,8]. The chemical flocculation method can make the oil droplets and suspended matter in wastewater quickly settle and flocculate, which is one of the main treatment methods for oilfield wastewater [9,10].

The organic polymer flocculant cationic polyacrylamide (CPAM) has the characteristics of a low additive amount, good turbidity removal and water purification effect, and high COD removal efficiency, and it has become the most commonly used polymer flocculant in the oilfield wastewater treatment process [11,12,13,14,15].

Polyacrylamide solid particles need to be prepared into a diluent of a certain concentration for use, which will undergo three stages of wetting, swelling, and dissolution during the dilution process [16]: wetting is the gradual solventization (hydration) of the surface of the polymer particles; swelling is the hydration of the polymer particles’ surface by the osmotic pressure of the solvent, during which small water molecules continue to penetrate into the polymer particles, resulting in the gradual swelling of the particles; and when the distance between the molecules within the swelling polymer particles reaches a certain value, the linear polymer molecular clusters begin to disperse, forming a solution, which is the process of dissolution. The dilution process of all water-soluble polymer solid particles is the same. The external fine particles will first quickly get wet, swell, and dissolve, forming a locally highly viscous solution, which will make a large number of solvated water molecules adhere to the outside of the polymer, preventing the internal small particles from further contact with the solvent water molecules, resulting in the external particles of the polymer being dissolved, while the internal particles will not even come into contact with the water molecules. These internal particles are “encapsulated” to make it difficult to reach the water molecules, forming a “fish-eye” that is difficult to dissolve. Once the “fish-eye” is formed, it will cause the waste of the flocculant, or it will block the pipeline and pump head, which will affect the production of the system. In addition, due to the slow dissolution rate of solid particle flocculants, a specialized device is needed to dissolve these flocculants, and the device operates continuously for 24 h, increasing equipment maintenance costs and energy consumption.

The solubility problem of CPAM solid particles has brought great trouble to the oilfield wastewater treatment process, restricting the improvement and innovation of the process. In recent years, researchers have begun to study the preparation technology of liquid cationic polyacrylamide products [17,18,19], but there are still many technical difficulties to be solved in these technologies, such as a low effective content, poor storage stability, poor water purification effect, complex preparation process, high production costs, etc. Liquid CPAM products represented by “water-in-oil” emulsions and “water-in-water” emulsions have received extensive attention from many researchers due to their fast dissolution rate [20,21,22,23,24], and the advantages and disadvantages of different products are shown in Table 1.

Franklin [25] prepared a polyacrylamide water-in-oil microemulsion, which had good stability and was not easy to separate. However, the viscosity of the product was relatively high. When the polymer content was 28.10%, the apparent viscosity of the microemulsion reached more than 321 mPa·s, and its mobility was too poor to be pumped in practical applications. Liu [26] prepared a water-in-water cationic polyacrylamide emulsion with a viscosity of 150~500 mPa·s, but its effective content was only about 15%. The preparation of this “water-in-water” emulsion required a strict monomer concentration. In the polymerization process, once the monomer concentration was too high, it was easy to form a gel rather than a stable emulsion. The “water-in-oil” emulsion prepared by Wang had a stable performance [27], no separation after 3 months at room temperature, and a dissolution time of about 10 min. However, the preparation process of a “water-in-oil” emulsion is very complicated, and it needs to be synthesized under the conditions of no metal ions and no oxygen, so the production cost is high and the cost performance of the product is relatively low. In short, the preparation technology of these liquid products is still immature, and there are obvious defects, such as a low effective content of the solid phase, with the effective content generally being no more than 30%, which brings great inconvenience to its transportation and production. Another disadvantage includes a poor storage stability, with long-term storage resulting in serious degradation. Finally, these liquid products have a low molecular weight, compared with solid particles and not only require greatly increasing the dosage of the agent, but at the same time, their flocculation and purification performance on wastewater is also poor and it is difficult to meet most of application requirements.

Due to the many defects of CPAM products existing in the current market, with for example, solid-type CPAM (CPAM particles) having the problems of a slow dissolution speed and easy formation into a “fish-eye”, while liquid-type CPAM products (water-in-water emulsions, water-in-oil emulsions, low-concentration aqueous solutions) have the problems of a low effective content, poor storage stability, expensiveness, and so on, the development of a novel type of CPAM flocculant with a fast dissolution speed, high effective content, and good storage stability is imperative. Therefore, the objective of this paper is to develop a novel type of CPAM, which, in comparison with the current work, has the following features: (1) good solubility, in that it can be quickly dissolved, not form a “fish-eye”, and solve the problems of CPAM waste, poor application effect, and pipeline blockage caused by the poor solubility of current solid CPAM products; (2) good stability, solving problems such as spoilage in summer, freezing in winter, and easy degradation of current liquid products; and (3) high effective content, reducing storage, transportation, packaging, and other costs, with the current liquid-type CPAM effective content being too low and emulsion-type CPAM being limited by its stability in the polymerization process, usually requiring a low concentration of monomers. Once a monomer concentration is too high, burst polymerization will occur a stable emulsion cannot be obtained. Meanwhile, solution-type CPAM is limited by its fluidity, and when a solution with a concentration of more than 10% is prepared, the solution will be too viscous and difficult to make flow.

In this paper, a novel type of liquid CPAM product, a CPAM suspension, was prepared, the optimal preparation process of the suspension was first studied, and then the dissolution performance, storage stability, flocculation, and water purification performance of the suspension were evaluated. Finally, a scale-up test was conducted to apply the produced CPAM suspension to oilfield wastewater treatment to verify the performance of the suspension for practical applications.

## 2. Materials and Methods

### 2.1. Materials and Instruments

Cationic polyacrylamide 4190 (CPAM 4190) was purchased from SNF China Co., Ltd., Taixing, China. #5 White Oil (#5WO) was purchased from Shenzhen Zhongruntong Chemical Co., Ltd., Shenzhen, China. Fumed silica (F-Silica 5000 mesh) was purchased from Yangshan Yuanfeng Powder Material Co., Ltd., Qingyuan, China. Organic bentonite (O-Bent) was purchased from CNPC Bohai Drilling Engineering Co., Ltd., Tianjin, China. Polyaluminum chloride (PAC) was purchased from Nanjing Xiangling Environmental Protection Technology Co., Ltd., Nanjing, China. Sodium dodecyl sulfate (SDS), sodium dodecyl benzene sulfonate (SDBS), polysorbate 80 (Tween 80), sorbitan tristearate 60 (Span 60), sorbitan tristearate 80 (Span 80), hexadecyl trimethyl ammonium bromide (CTAB), and dodecyl phenol polyoxyethylene ether 10 (OP-10) were purchased from Beijing Yili Fine Chemicals Co., Ltd., Beijing, China. Except for CPAM 4190, #5WO, F-Silica, and O-Bent, which were industrial-grade, the other reagents were analytical-grade.

The six-speed rotary viscometer, ZNN-D6, and digital display high-speed mixer, GJ-2S, used were manufactured by Qingdao Tongda Special Instrument Co., Ltd., Qingdao, China. The multi-functional high-speed blender, SUS-304, employed was manufactured by Wuyi Hainan Electric Co., Ltd., Haikou, China. The standard sample sieve used was manufactured by Beijing Chemical Glass Station Bioanalytical Technology Co., Ltd., Beijing, China. The desktop high-speed centrifuge was manufactured by Changsha Dongwang Experimental Instrument Co., Ltd., Changsha, China. The high-speed adjustable homogeneous emulsifier, FSV-2, was manufactured by Changzhou Jintan Jingda Instrument Manufacturing Co., Ltd., Changzhou, China. The digital display laser turbidity meter SGZ-2 was manufactured by Shanghai Yuefeng Instrument Co., Ltd., Shanghai, China. The optical microscope, CX-43, was manufactured by Olympus (China) Co., Ltd., Beijing, China.

### 2.2. Preparation Method of CPAM Suspension

Firstly, the industrial-grade CPAM solid particles were crushed by a pulverizer for 2 min and then poured out, the crushed CPAM solid particles were then sieved through a 100-mesh standard sample sieve, and a certain amount of separating agent was added and mixed well with a stirrer to obtain CPAM fine powder for use.

Then, a certain amount of rheology modifier was added into the oil phase solvent, and the solution was emulsified and stirred for 2 min with a high-speed homogenizer to obtain the suspension solvent.

**Figure 1:**
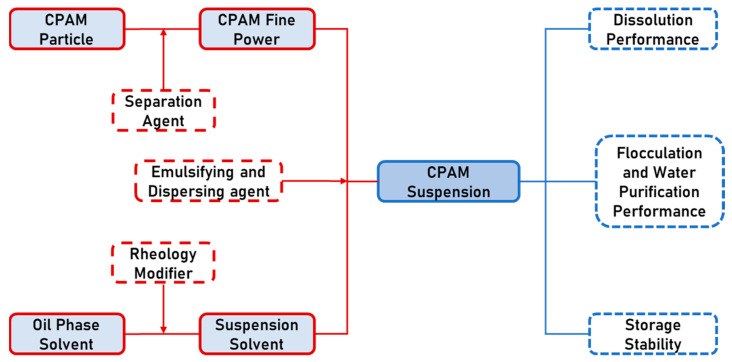
Schematic diagram of the CPAM suspension preparation.

Finally, the CPAM fine powder was added with the emulsifying and dispersing agent together into the suspension solvent and emulsified and dispersed at a high speed for 10 min with a high-speed homogenizer to obtain a stable CPAM suspension. The preparation schematic is shown in Figure 1.

### 2.3. Performance Evaluation Methods for Suspension

#### 2.3.1. Evaluation Method of Solubility

Non-Newtonian fluid rheological performance test—A certain volume of non-Newtonian fluid was taken, a special measuring cup was filled by it for viscosity testing, the readings of the six-speed rotational viscometer at different speeds were recorded, and the following formulas were used to calculate the rheological parameters:

AV = R_600_/2 (1)

PV = R_600_ − R_300_ (2)

YP = 0.5(R_300_ − PV) (3)

where

R_600_ = the reading of the viscometer at 600 r/min (dia);

R_300_ = the reading of the viscometer at 300 r/min (dia);

AV = apparent viscosity (mPa·s);

PV = plastic viscosity (mPa·s);

YP = yield point (Pa).

Dissolution time test—Under the speed of 200 rpm, a certain mass of suspension was slowly added dropwise to the beaker containing deionized water, and the apparent viscosity value of the solution was recorded at intervals until the apparent viscosity value no longer increased, which was the dissolution time for the sample to fully form an aqueous solution.

#### 2.3.2. Evaluation Method of Stability

A certain volume of CPAM suspension was placed in a test tube and the height from the bottom of the test tube to the liquid surface was recorded as L_1_. After storing the test tube at room temperature for 1 day, 3 days, and 7 days, the height of the separated supernatant was recorded as L_2_, and the following formula was used to calculate the storage separation rate of the suspension:

S = L_2_/L_1_ × 100% (4)

where

S = storage separation rate (%);

L_1_ = height of the suspension (cm);

L_2_ = height of separated supernatant (cm).

A certain mass of CPAM suspension was weighed and recorded as M_1_, poured into a centrifuge tube and sealed, placed into a high-speed centrifuge, and then centrifuged at 2000 rpm for 30 min. The mass of the centrifuged supernatant was weighed and recorded as M_2_, and the following formula was used to calculate the centrifugal separation rate of the suspension:

C = M_2_/M_1_ × 100% (5)

where

C = centrifugal separation rate (%);

M_1_ = height of the suspension (g);

M_2_ = height of separated supernatant (g).

#### 2.3.3. Evaluation Method of Flocculation and Purification Performance

The simulated wastewater preparation—A certain mass of diatomite was weighed, a certain volume of tap water was added, and a high-speed mixer was used to mix the simulated wastewater well with a turbidity of about 1000 NTU. The oilfield wastewater was taken from the combined wastewater treatment stations of the oilfield of PetroChina Daqing, including La-I-1, Xing-1, and Xing-20.

Wastewater turbidity removal rate test—A turbidimeter was first calibrated with 400 NTU standard solution. After the reading of the turbidimeter found it to be stable, a certain volume of wastewater was taken and put into the test bottle of the turbidimeter. The original turbidity of the wastewater was recorded as U_1_. A certain amount of flocculant was added to the wastewater, stirred thoroughly, and it was allowed to stand for 2 min. The upper clear liquid was then taken and its turbidity was tested as U_2_. The following formula was used to calculate the turbidity removal rate of the flocculant on the wastewater:

T = U_2_/U_1_ × 100% (6)

where

T = turbidity removal rate (%);

U_1_ = turbidity of original wastewater (NTU);

U_2_ = turbidity after turbidity removal (NTU).

#### 2.3.4. Determination of Oil Content

In this study, the oil content in the wastewater was determined using the spectrophotometric method [28], whereby the oily wastewater (from Daqing Bluestar Environmental Protection Engineering Co., Ltd. (Daqing, China) wastewater treatment station) was placed in a dispensing funnel, a certain amount of gasoline and hydrochloric acid was added to extract the oil in the wastewater, the extract was dehydrated by anhydrous calcium chloride and then filtered, the filtrate was evaporated to remove the gasoline in the water bath at 80 °C, and the standard oil samples were obtained to be used.

The standard oil sample was used to prepare the standard oil solution with an oil concentration of 5.00 mg/mL, and then 0.00 mL, 0.50 mL, 1.00 mL, 1.50 mL, 2.00 mL, 5.00 mL, and 10.00 mL of the standard oil solution was put into a 50 mL colorimeter tube. Then, the absorbance was tested by a spectrophotometer (λ = 430 nm), and the relationship between the oil content and absorbance was obtained, as shown in Table 2. A standard curve was calculated by the least squares method: y = −0.27 + 42.11x.

Hydrochloric acid and gasoline were added to a certain volume of wastewater for extraction, the extracted oil phase was put in a colorimeter tube, gasoline was added to dilute it to the scale, and a spectrophotometer was used to test its absorbance. To find out the oil content on a standard curve, the oil content calculation formula used was the following:

O = M_o_/V_o_ × 10^3^ (7)

where

O = oil content (mg/L);

M_o_ = oil content found on the standard curve (mg);

V_o_ = volume extracted from wastewater (mL).

#### 2.3.5. Optical Microscope

Slides and coverslips were first cleaned using ethanol and then dried in an oven at 85 °C. Samples of the CPAM suspension were taken with a dropper, a drop was placed on a slide and covered by a coverslip, and the suspension sample was observed using the optical microscope Olympus CX-43 at different magnifications.

## 3. Results and Discussion

### 3.1. Influencing Factors of the Suspension

#### 3.1.1. Effect of the Separating Agent

**Figure 2:**
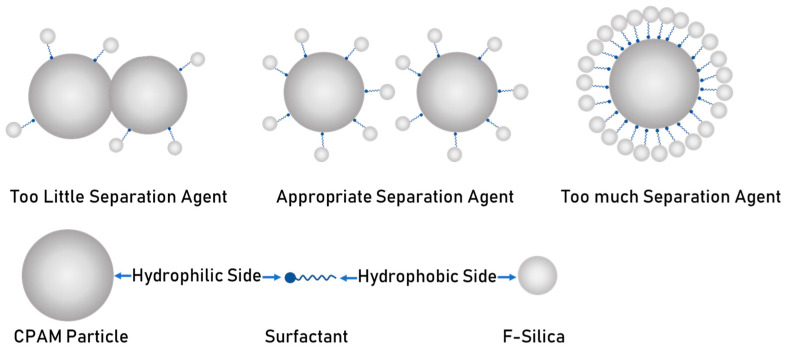
The adsorption mechanism of F-Silica.

The finer polymer particles are, the easier it is for them to agglomerate, so to prevent the fine CPAM particles in the water from forming a “fish-eye”, it was necessary to add a separating agent to disperse the particles. F-Silica [29,30] has the characteristics of a small particle size, large specific surface area, and strong adsorption, which can be effectively adsorbed on the surface of CPAM particles, making the suspension more stable [31]. The adsorption mechanism of F-Silica is shown in Figure 2.

F-Silica is prepared using a fumed phase method, and the surface of its particles contain silicon–oxygen bonds, which are hydrophobic, while the surface of CPAM particles contain hydrophilic groups such as amine groups, which are hydrophilic. The suspension contains surfactants with an amphoteric molecular structure; with hydrophilic groups at one side and hydrophobic groups at the other side, the hydrophilic side can be attached to the surface of CPAM particles, while the hydrophobic side can be in contact with the surface of the F-Silica. Therefore, the surfactant can be used as a connecting thread to link the two types of particles, F-Silica and CPAM, and this is the mechanism by which F-Silica adsorbs on the surface of CPAM particles. If the amount of F-Silica is too little, it cannot play a role in separation, and the CPAM particles will be in contact with each other; if the amount of F-Silica is too much, it will cover all the CPAM particles, which is not conducive to dissolution, so the amount of F-Silica should be controlled in an appropriate range.

**Figure 3:**
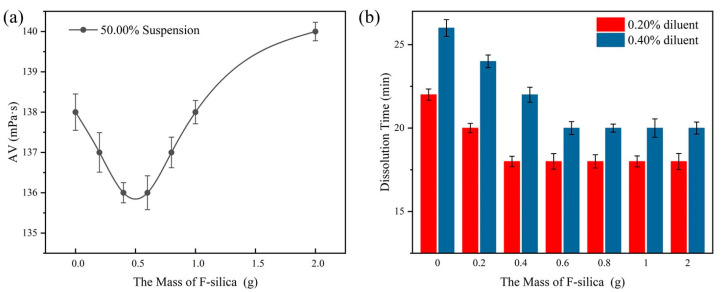
Effects of F-Silica: (**a**) trend of the apparent viscosity of the suspension (at 50% effective content of the suspension); (**b**) trend of dissolution time (dilution concentrations of 0.20% and 0.40%).

In this study, different masses of F-Silica were added to the suspension to test the apparent viscosity of the suspension and the dissolution time of different concentrations of diluent, and the results are shown in Figure 3.

As can be seen from Figure 3a, the apparent viscosity of the suspension showed a trend of decreasing and then increasing with the increase in the mass of F-Silica, and the apparent viscosity of the suspension reached a minimum of 136.00 mPa·s at its addition of 0.40 g/60.00 g. From Figure 3b, it can be seen that by adding 0.40 g/30.00 g of F-Silica, the dissolution time of 0.20% diluent was shortened from 22.00 min to 18.00 min. However, the dissolution time was not further shortened by the addition of F-Silica more than 0.40 g/30.00 g. This is because with the addition of F-Silica, the overall structure of the CPAM particles in the suspension was fluffier and easier to flow, but with the excessive addition of F-Silica, the internal friction between particles increases, which makes it difficult for the particles to slide into the suspension, thus increasing the apparent viscosity of the suspension. The presence of F-Silica creates a barrier between the polymer’s fine particles, which renders it more difficult to agglomerate in water and more conducive to dissolution. F-Silica adsorbed on the surface of the polymer particles only plays a segregation role, and adding too much is not conducive to the diffusion of particles in water, so the amount of F-Silica additives selected as 0.40 g/60.00 g was appropriate.

#### 3.1.2. Effects of the Emulsifying and Dispersing Agent

In this study, WO was used as the solvent of the suspension, but WO could not be dissolved in water, so it was necessary to add surfactants with hydrophilic and non-hydrophilic groups to the suspension so that WO could be quickly dispersed and dissolved in water, and at the same time, to promote the rapid diffusion of the polymer’s fine particles in water.

**Figure 4:**
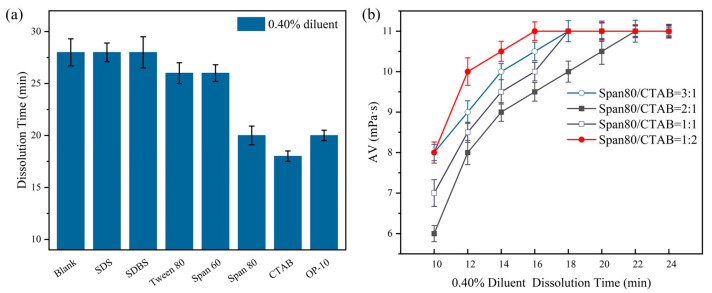
Effects of emulsifying and dispersing agent: (**a**) dissolution time (dilution concentration of 0.40%); (**b**) synergistic effect of CTAB and Span 80 (dilution concentration of 0.40%).

Both the type and amount of surfactant added affect the emulsification and dispersion of WO. In this study, 1.00 g/60.00 g of different types of surfactants was added to the suspension and then the complete dissolution time of the suspension in water was tested; the results are shown in Figure 4.

As shown in Figure 4a, it can be seen that the emulsification effects of SDS and SDBS on WO were not good, and they did not emulsify or disperse WO in water, resulting in no improvement in the dissolution time of the suspension. Meanwhile, Tween 80 and Span 60 had some emulsification effect, which could accelerate the dissolution of the suspension to a certain extent. The dissolution time of the suspension in water was obviously accelerated, and the shortest dissolution time was 18.00 min. Since the use of OP emulsifiers is restricted by relevant regulations due to their harmful effects on the environment [32], CTAB and Span 80 were used as the main and auxiliary emulsifiers for the suspension from the perspective of environmental friendliness.

After Span 80/CTAB was added to the suspension at different mass ratios, the dissolution time of the 0.4% diluent was tested and the results are shown in Figure 4b. It can be seen that when the mass ratio of Span 80:CTAB was 1:1, the apparent viscosity of the diluent reached a maximum value of 11.00 mPa·s in the shortest time, which took only 16.00 min. This is because under the synergistic effect of Span 80 and CTAB, after the suspension was added to water, WO molecules wrapped around the surfaces of the fine CPAM particles; they rapidly emulsified and dispersed in water because the surfaces of the polymer particles came into rapid contact with water molecules to achieve rapid dissolution.

#### 3.1.3. Effects of the Rheology Modifier

**Figure 5:**
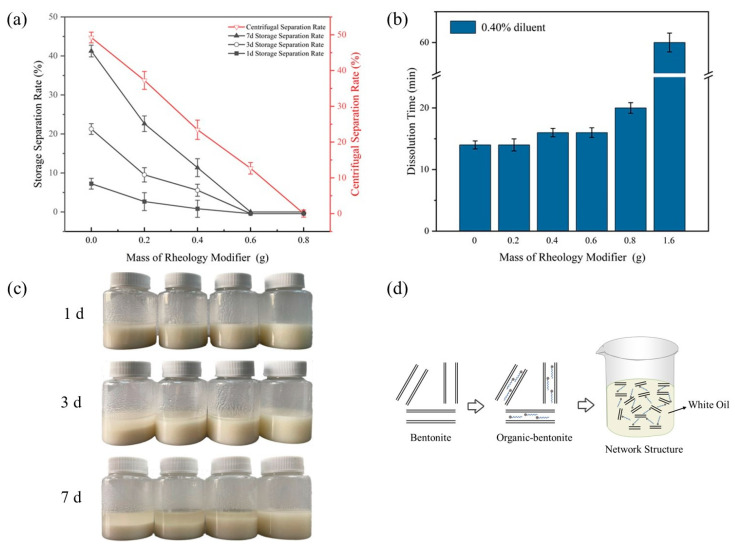
Effects of the rheology modifier: (**a**) suspension separation rate (centrifuged at 2000 rpm for 30 min); (**b**) dissolution time (dilution concentration of 0.40%); (**c**) room temperature storage; (**d**) mechanism of shear force enhancement.

The density of the WO used in this study was about 0.85 g/cm^3^, while the density of the CPAM particles was about 1.32 g/cm^3^, and the polymer particles added to the WO directly settled, so it was necessary to improve the shearing force of the WO. In the field of oilfield chemical drilling, bentonite is modified into organic bentonite (O-Bent), which can be dispersed and swelled in WO to form a spatial network structure and increase the viscosity and shearing force of oil-based drilling fluids (Figure 5d), which is used to enhance the cutting transportation of drilling fluids [23,24,25,26,27,28,29,30,31,32,33,34,35]. Therefore, in this study, different masses of O-Bent were first added to the suspension as a rheology modifier, and then the rheology and separation rate of the suspension were tested. The experimental results are shown in Table 3 and Figure 5.

As can be seen from Table 3, with the increase in the addition of O-Bent, the viscosity and dynamic shear of the suspension were significantly improved, but when the mass of the rheology modifier was 1.60 g/60.00 g, the suspension became gelatinous and difficult to make flow. It was difficult for it to disperse after adding it to the water, which indicates that the addition of a rheology modifier should be sparing. As can be seen from Figure 5a, when the mass of the rheology modifier was 0.60 g/60.00 g, the suspension was able to be stored for 7 days without separation, which indicates that when stored at room temperature, O-Bent can provide a solvent with enough shear force so that the solvent can suspend CPAM particles. When the mass of the rheology modifier was 0.80 g/60.00 g, the suspension did not separate when stored at room temperature for 7 days, nor did it separate when centrifuged at 2000 rpm for 30 min, but the apparent viscosity of the suspension was higher. The diffusion of the suspension was slower after the drop was added to the water, resulting in a longer time for the complete dissolution of the suspension in the water (as shown in Figure 5b), and given the comprehensive consideration of its stability and dissolvability, the mass of the rheology modifier being 0.60 g/60.00 g was more appropriate.

**Figure 6:**
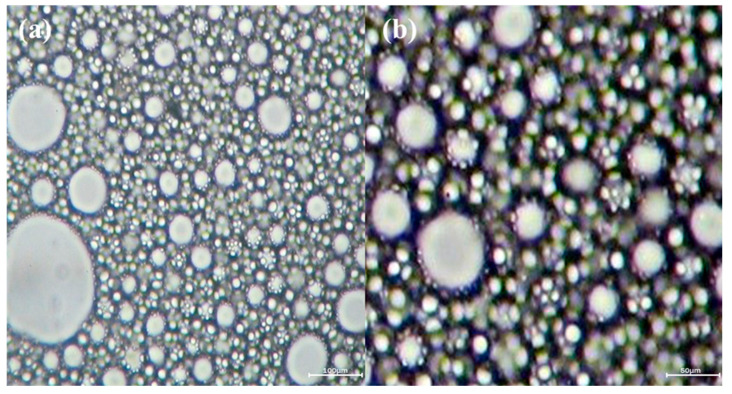
Image of the suspension under an optical microscope: (**a**) 200× magnification; (**b**) 400× magnification.

As can be seen from Figure 6, the size of most polymer particles in the suspension was <100 μm and the polymer particles were uniformly distributed in the suspension, with obvious boundaries between the particles. Furthermore, there was no aggregation of the particles to precipitate, which indicates that the rheology modifier played a role in improving the shear force, so that the suspension had enough shear force to suspend the polymer particles. In addition, it is obvious that there were many fine particles attached to the surface of the polymer particles, which indicates that the separating agent F-Silica was indeed adsorbed at the surface of the polymer particles, making the polymer particles dispersed from each other.

Considering all these influencing factors, the proportions of each component of the suspension were obtained as shown in Table 4, and the suspension prepared according to these proportions can have good suspension stability and be completely dissolved in a short time.

### 3.2. Comparison of the Products’ Performance

#### 3.2.1. Comparison of the Dissolution Performance

In this study, original particles of CPAM, fine particles of the CPAM crushed to a 100 mesh, and a 50% content of CPAM suspension were prepared at different concentrations of diluent. The rheology and complete dissolution time of the diluents were tested, and the results are shown in Table 5.

As can be seen from Table 5, CPAM fine particles easily form a “fish-eye” and are difficult to dissolve in water no matter how much concentrated diluent is prepared; at 0.40% diluent, the AV value began to decline, but the formed “fish-eye” still failed to dissolve completely. The original CPAM particles started to resemble a “fish-eye” when the concentration reached 0.40%, making the dissolution time more than 120.00 min; benefiting from the liquid form of the product, the CPAM suspension can be quickly dispersed in water, and the CPAM particles have a smaller particle size, so the dissolution time is greatly shortened (0.20% diluent dissolved in 12.00 min). Since the CPAM suspension needs to be mechanically refined during the preparation process, this process will break part of the molecular chain of the linear polyacrylamide, which will lead to a certain degree of viscosity decrease in the diluent. In the preparation of 0.20% CPAM diluent, compared with the original particles, although the apparent viscosity of the suspension decreased by 12.50%, the dissolution time could be shortened by 83.33%, and did not produce a “fish-eye”; the decrease in apparent viscosity was worthwhile.

#### 3.2.2. Comparison of Flocculation and Water Purification Performance

**Figure 7:**
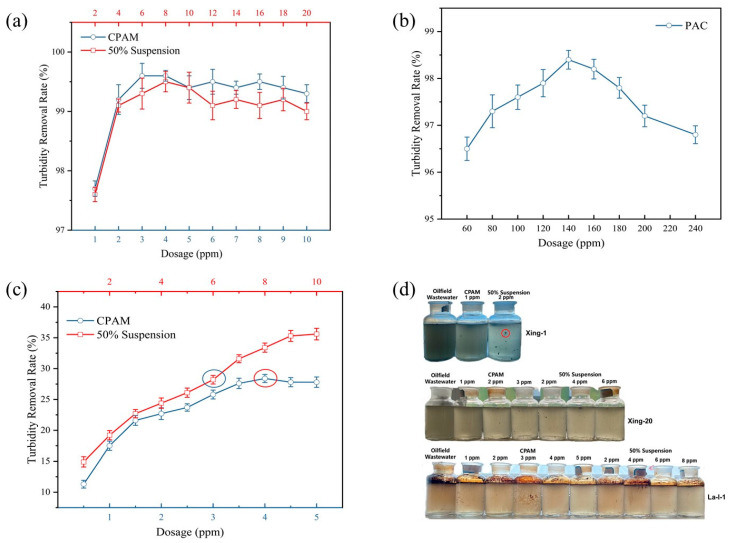
Comparison of flocculation and water purification performance of different products: (**a**) simulated wastewater (organic polymer flocculant, dilution concentrations of 0.20%); (**b**) simulated wastewater (inorganic flocculant, dilution concentrations of 10.00%); (**c**) oilfield wastewater (dilution concentrations of 0.20%); (**d**) water purification effect.

In this study, we prepared original CPAM particles into a diluent with a concentration of 0.10%; the CPAM suspension, into a diluent with a concentration of 0.20%; and the inorganic flocculant PAC, into a diluent with a concentration of 10.00%. Then, the prepared diluent was added to the simulated wastewater and oilfield wastewater according to different dosing amounts, the turbidity before and after dosing was tested, and then the turbidity removal rate was calculated, as shown in Figure 7.

As can be seen from Figure 7a, for the diatomite-simulated wastewater, the best turbidity removal effect was achieved at a polymer dosing of 4.00 ppm, and the turbidity removal effects of the original CPAM original particles and the CPAM suspension were basically the same, with optimal turbidity removal rates of 99.60% and 99.50%, respectively. Conversely, the inorganic flocculant PAC at a 140.00 ppm dosage showed the best turbidity removal effect for simulated wastewater and the turbidity removal rate was 98.40% (Figure 7b), which was lower than the CPAM suspension. For the oilfield wastewater (Xing-1 combined wastewater treatment station), the turbidity removal rate of the CPAM suspension was higher than that of the original CPAM particles under the same polymer dosage, with the optimal turbidity removal rate of the CPAM suspension being 35.60%, and that of the original CPAM particles, being 28.40% (Figure 7c). When the dosage of the CPAM suspension was 1.5 times the optimal dosage of the original CPAM particle, the turbidity removal rates were basically the same, with 28.40% and 28.20%, respectively. This is due to the addition of cationic surfactants in the suspension, as cationic surfactants can assist the polyacrylamide adsorption of suspended particles in wastewater through electrostatic action, whereby the more negatively charged suspended particles are in wastewater, the more pronounced is this auxiliary flocculation effect [36]. As shown in Figure 7d, for the oilfield wastewater from the Xing-1 combined wastewater treatment station, the wastewater with the addition of the CPAM suspension was obviously clearer and brighter than that with the addition of the original CPAM particles, and the flocs aggregated out of the wastewater were obviously larger.

#### 3.2.3. Comparison with Other Study

In this study, we compared the performances of a CPAM suspension with the current liquid CPAM study of the same type, and the results are shown in Table 6.

As can be seen from Table 6, the effective content of the suspension can reach 50.00%, which is higher than the emulsion-type CPAM (28.10% and 30.00%, respectively); although the emulsion-type CPAM has the advantage of a better dissolution speed, due to its preparation process being more complex, the high requirements for its instrumentation, and its subsequent preparation cost being high, the market price of the emulsion with a content of 30% is about CNY 3000.00/t, while the market price of the 50% content suspension is about CNY 11,500.00/t, so the CPAM suspension is more cost-effective.

### 3.3. Application

#### 3.3.1. Background of the Wastewater Station

**Figure 8:**
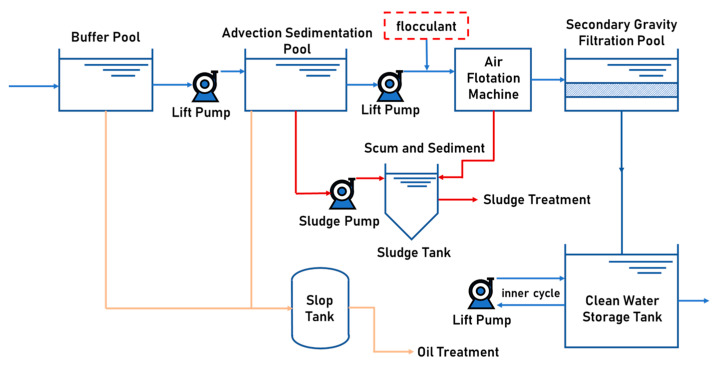
The wastewater treatment process for the wastewater station.

We conducted a scale-up test and produced 108.00 kg of CPAM suspension, which was applied to the wastewater treatment station of Daqing Bluestar Environmental Protection Engineering Co., and the wastewater treatment process at the wastewater station is shown in Figure 8.

As shown in Figure 8, there is a buffer pool in the wastewater station for buffering incoming water, with a design volume of 150 m^3^, steel mixed structure, internal corrosion, and seepage prevention, with a mud hopper at the bottom and oil scraping equipment on the surface of the pool. There are two advection sedimentation pools, the sedimentation part of the effective water depth is 3.0 m, the sedimentation part of the effective volume is 270 m^3^, the pool length is 20.0 m, and the pool width is 4.5 m. The sludge tank volume is 35 m^3^, the internal setup consists of a scum scraper and sediment scraper, and the sludge discharge mode for the use of the bottom of the sludge pump involves a sludge hopper that emits intermittent sludge discharge. There are two air flotation machines in the station, and the displacement of the air flotation machine lifting pump is 50 m^3^/h. The CPAM suspension flocculant diluent is added at the inlet of the air flotation machine. After the suspended matter in the wastewater is flocculated, the flocculant will float and be scraped away by the scum scraper at the top of the air flotation machine. There are eight secondary gravity filtration pools, the effective volume of the sedimentation part is 216 m^3^, and the length, width, and height of the tanks are all 3.0 m.

#### 3.3.2. Application Effects

**Figure 9:**
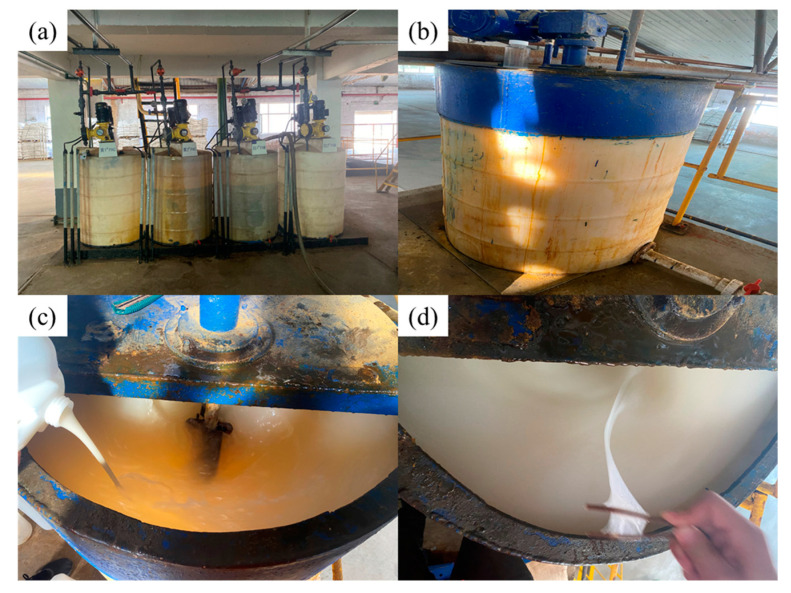
Field dosing situation: (**a**) CPAM particle dosing tank; (**b**) CPAM suspension dosing tank; (**c**) CPAM suspension for dilution; (**d**) after full dissolution.

The CPAM suspension with a concentration of 1.20% added to a 500 L dosing tank for dilution, with stirring for 20 min to fully dissolve it, and then use of a dosing pump with a displacement of 500 L/h of dosing. The specific dosing parameters are shown in Table 7 and the field dosing equipment is shown in Figure 9.

As can be seen from Figure 8, the CPAM suspension dissolved very well in the dosing tank, at a concentration of 1.20%, the suspension could be uniformly dispersed in the water, and it could be completely dissolved within 20 min, without a “fish-eye” situation. We observed the flocculation of the wastewater in the air flotation machine before and after dosing, as shown in Figure 9. After the completion of dosing, wastewater samples were taken at the outlet to test their turbidity and oil content, and the experimental results are shown in Table 7.

**Figure 10:**
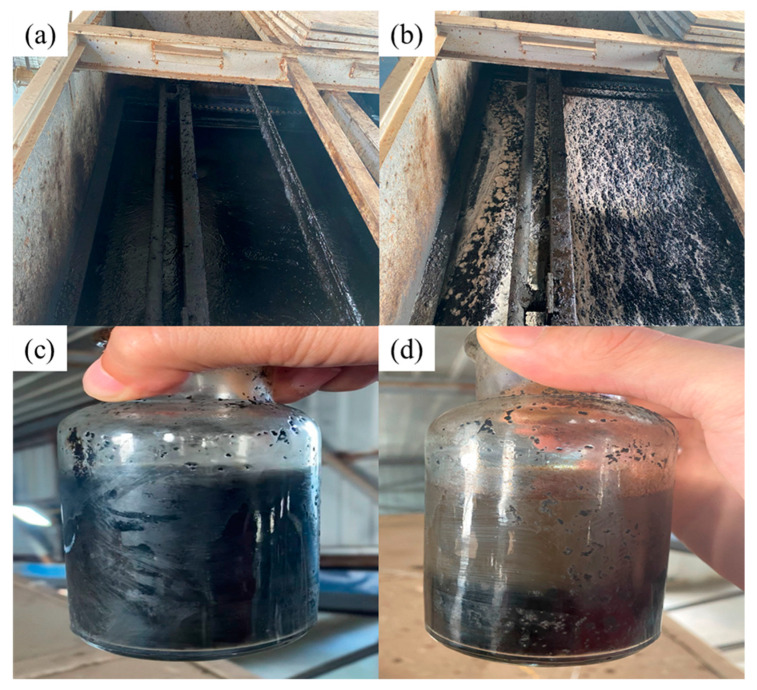
Wastewater before and after field dosing: (**a**) wastewater in the air flotation machine before dosing; (**b**) wastewater in the air flotation machine after dosing; (**c**) wastewater at the outlet before dosing; (**d**) wastewater at the outlet after dosing.

As can be seen from Figure 10, in the air flotation machine, no obvious flocs were floating in the wastewater before dosing, while after dosing, the wastewater had obvious, large, and dense floating flocs; in the air flotation machine outlet, the wastewater in the bottle after dosing was significantly clearer, and there was an obvious layer of flocs settling at the bottom of the bottle. From Table 8, it can also be seen that after treatment with the CPAM suspension flocculant, the turbidity and oil content of the wastewater were significantly reduced, the turbidity was reduced from 1126.00 NTU to 554.62 NTU (turbidity removal rate of 50.74%), and the oil content was reduced from 68.55 mg/L to 20.36 mg/L (oil removal rate of 70.30%). Meanwhile, after treatment with CPAM particles, the turbidity of the wastewater was reduced to 756.23 NTU (turbidity removal rate of 32.84%), and the oil content was reduced to 27.24 mg/L (oil removal rate of 60.26%), which indicates that the flocculation and oil removal effects of the CPAM suspension on wastewater were better than those of the CPAM particles.

## 4. Conclusions

A CPAM suspension prepared from CPAM solid particles can reach an effective content of 50%, apparent viscosity of 136.00 mPa·s and show no separation after 7 days of storage at room temperature, as well as no separation after 30 min of centrifugation at a speed of 2000 rpm. When diluted in a 0.40% aqueous solution, compared with CPAM solid particles, its apparent viscosity decreased by 12.5%, while its dissolution time was shortened by 81.40%, which was only 16.00 min.

The optimal composition ratio of the CPAM suspension was 50.00% CPAM fine powder, 46.87% oil phase solvent, 0.63% separating agent, 1.56% emulsifying and dispersing agent, and 0.94% rheology modifier.

The best turbidity removal rate of the CPAM suspension for diatomite-simulated wastewater was 99.50%, slightly lower than the CPAM solid particles of the best turbidity removal rate of 99.60%, but better than the inorganic flocculant PAC’s best turbidity removal rate of 98.40%. The best turbidity removal rate of the CPAM suspension for oilfield wastewater was 35.60%, which is better than the best turbidity removal rate of the CPAM solid particles at 28.40%; when an additional amount of CPAM suspension was 1.5 times the additional amount of CPAM solid particles, the same turbidity removal and purification effect could be achieved.

In the scale-up test, the CPAM suspension, formulated with a 1.20% concentration, could be completely dissolved within 20 min; the CPAM suspension was superior to the CPAM particles in flocculation and the oil removal of wastewater, with the turbidity removal rate of the CPAM suspension being 50.74% and the oil removal rate being 70.30%, while the turbidity removal rate of the CPAM particles was only 32.84% and the oil removal rate was 60.26%.

## Figures and Tables

**Table 1:** Comparison of advantages and disadvantages of different CPAM products.

CPAM Product	Advantages	Disadvantages
Solid particles	Convenient transportation, stable storage	Slow dissolving speed, easy to form fish-eye
Low-concentration solution	Fast dissolving speed, convenient preparation	Inconvenient transportation, low effective content (≤10%), easy to deteriorate, odor
Water-in-water emulsion	Fast dissolving speed	Easy to separate layers, easy to degrade
Water-in-oil emulsion	Fast dissolving speed, good stability	Low effective content (≤30%), high price, complex preparation processes
Suspension (this work)	No fish-eye, high effective content, simple preparation process	Dissolving speed is relatively slow, loss of solution viscosity

**Table 2:** Relationship between oil content and absorbance.

Oil Content y (mg)	Absorbance x
0	0
2.50	0.062
5.00	0.123
7.50	0.187
10.00	0.248
25.00	0.613
50.00	1.186

**Table 3:** Effects of the rheology modifier on the suspension.

Mass of Rheology Modifier (g)	R_600_ (dia)	R_300_ (dia)	AV (mPa·s)	PV (mPa·s)	YP (Pa)
0.00	203.00	104.00	101.50	99.00	2.50
0.20	230.00	120.00	115.00	110.00	5.00
0.40	251.00	132.00	125.50	119.00	6.50
0.60	274.00	147.00	137.00	127.00	10.00
0.80	296.00	162.00	148.00	134.00	14.00
1.60	>300.00	177.00	>150.00	>123.00	>27.00

**Table 4:** Percentages of components in the suspension.

Function	Component	Mass (g)	Percentage (%)
Flocculant	CPAM fine powder	32.00	50.00
Solvent	#5WO	30.00	46.87
Separating agent	F-Silica	0.40	0.63
Emulsifying and dispersing agent	Span 80	0.50	0.78
	CTAB	0.50	0.78
Rheology modifier	O-Bent	0.60	0.94

**Table 5:** Comparison of dissolution performances of different samples.

CPAM Sample	Concentration (%)	AV (mPa·s)	PV (mPa·s)	YP (Pa)	Dissolution Time (min)
Original particle	0.10	6.50	6.00	0.50	64.00
	0.20	12.00	12.00	0.50	96.00
	0.40	30.00	28.00	2.00	Fish-eye, 120.00+
Fine particle	0.10	5.00	6.00	0.00	Fish-eye, 86.00
	0.20	10.00	9.00	1.00	Fish-eye, 120.00+
	0.40	27.50	26.00	1.50	Fish-eye, 120.00+
Suspension (50%)	0.20	5.50	5.00	0.50	12.00
	0.40	10.50	10.00	0.50	16.00
	0.80	29.00	27.00	2.00	22.00

**Table 6:** Comparative data from the CPAM suspension and other studies.

CPAM Sample	Effective Content (%)	Molecular Mass (million)	Dissolution Time (min)	Apparent Viscosity (mPa·s)	Market Price (CNY/t)
Particle	100.00	12.32	96.00	–	15,000.00
Suspension	50.00	11.57	16.00	136.00	11,500.00
Water-in-oil emulsion [25]	28.10	4.50~6.50	10.00	321.00	–
Water-in-water emulsion (a Chinese brand)	30.00	0.35	6.00	108.00	30,000.00

**Table 7:** Dosing parameters.

CPAM Sample	Wastewater Treatment Capacity (m^3^/d)	Dosing Concentration (mg/L)	Daily Dosage (kg)	Dosing Days (d)	Total Dosage (kg)
Particle	300.00	60.00	18.00	1	18
Suspension		120.00	36.00	3	108

**Table 8:** Wastewater quality indicator testing.

Wastewater Sample		Turbidity (NTU)	Oil Content (mg/L)
Original		1126.00	68.55
After adding CPAM particles		756.23	27.24
After adding CPAM suspension	First day	536.21	18.36
	Second day	569.32	22.68
	Third day	558.34	20.04
	Average	554.62	20.36

## Data Availability

Data are available from the authors upon request.
